# The Importance of Individual and Expert Knowledge Grows as Clan Identity Diminishes: The Bedouin of Southern Israel Adapt to Anthropocene Ecology

**DOI:** 10.3390/jintelligence13050051

**Published:** 2025-04-23

**Authors:** Michael Weinstock, Turky Abu Aleon, Patricia M. Greenfield

**Affiliations:** 1 School of Education, Ben-Gurion University of the Negev, Be’er Sheva 8410501, Israel; turkyabu@gmail.com; 2 Department of Psychology, University of California, Los Angeles, CA 90095, USA; greenfield@psych.ucla.edu

**Keywords:** epistemic perspective, socioecological change, Bedouin, clan identity, extended-family identity, local identity, parents’ education, media use

## Abstract

Before the Anthropocene, Bedouin communities in Southern Israel were based on a clan structure—a kin-based social network; clans were culturally and socially homogenous communities with a strong authority structure. Work consisted of subsistence activities necessary for physical survival. Group-based authority and cooperative problem solving were adaptive in this ecology. Throughout the Anthropocene, the Bedouin of Southern Israel have had to adapt to diverse urban environments, expanded educational opportunity, and exposure to media emanating from different cultures. Our study explored the implications of these ecological shifts for epistemic thinking by comparing three generations of 60 Bedouin families: adolescent girls, their mothers, and their grandmothers (*N* = 180). Families were evenly divided among three residence types differing in degree of urbanization and degree of population homogeneity: unrecognized Bedouin villages consisting of single clans; recognized Bedouin villages, towns, or cities, consisting of multiple clans; and ethnically diverse cities. Results: Across the generations, media exposure and formally educated parents have weakened the epistemic authority of family elders, in turn weakening clan identity. Ethnically diverse cities have weakened extended family identity. At the same time, personal knowledge and professional expertise have gained new cultural importance. These changes in epistemology and identity are adaptive in the ecological environments that have multiplied in the Anthropocene era. Local identity was strongest both in diverse cities, with their many attractions, and in unrecognized villages, where the population continues to occupy ancestral lands.

## 1. Introduction

In the current research, we examine shifts in Bedouin epistemology that have taken place during the Anthropocene. Bedouin culture has undergone notable changes over the last few generations from one very much rooted in a culture predating the Anthropocene to one that has had to increasingly adapt to ecologies in which there is greater exposure to formal education, media technology, and urban environments. These three intertwined features are characteristics of both Gesellschaft ecologies, which were first described by the German Sociologist Tönnies (1887/1999), and of the Anthropocene (Preiss 2022). In contrast, before the Anthropocene, Israeli Bedouins learned subsistence skills, like herding, from older generations of the family, communicated through in-person interaction, and lived in small clan-based villages. These are intertwined features of Gemeinschaft ecology (Tönnies 1887/1999). The German terms are used to indicate two ecological syndromes with interrelated features (Greenfield 2009).

Luria, collecting his data in Uzbekistan in 1931, was probably the first to demonstrate the cognitive effects of exposure to even limited formal education (Luria 1976); he found that reasoning moved away from a focus on social utility as it became more abstract. In Senegal, Greenfield (1966) found that, among the Wolof, schooling reduced children’s attention to an authority figure in a cognitive task and increased their attention to their own actions and thought processes. Correa-Chavez (2016) found less collaborative problem solving and more individual problem solving among Mexican heritage children whose parents had more formal education and less recent connection with rural practices. Hence, the Gesellschaft feature of schooling led to a more individualistic style of cognition; it decreased cognitive reliance on authority figures and the group, a style typical of people who have learned practical skills at home and in the community (Lancy 2012). Our research group has shown how movement from more Gemeinschaft to more Gesellschaft ecologies, the dominant direction of social change across the globe, produces still other cognitive shifts in the individualistic direction, for example, the rise of personal opinions in Romania as a function of more diverse media experience (Ionescu et al. 2023). Under rapid social change, experts (such as pediatricians and psychologists) replace family members (e.g., parents) as child development experts (El-sana et al. 2024; Keller et al. 1984). In the present research, we explore how multiple shifts in the Gesellschaft direction—formal education, media exposure, and diverse urban environments—elevate Gesellschaft-adapted approaches to knowledge: Both personal knowledge and expert knowledge gain increasing value, while the epistemic authority of older family members and group-based identities both decline in influence.

### 1.1. Moving Away from a Social Structure Based on Clans and Large Extended Families

During the Anthropocene, there have been external forces impinging on the traditional clan-based social structure. These forces have produced shifts in the absolute primacy of clans in the social structure and in their exclusive power. (Sometimes these clans are called “tribes” in the social science literature (e.g., Allassad Alhuzail 2022)). In terms of social relations, each clan was isolated from other clans. In addition, the large, interdependent extended family was also an important unit within a clan. Both these family structures are characteristics of high kinship intensity (Bahrami-Rad et al. 2022; Henrich 2020).

Traditionally a nomadic people who lived primarily by rearing sheep and camels in the desert, the Bedouin have become increasingly semi-nomadic and settled because of the demands of administrative non-Bedouin governments, first with the Ottomans in the second half of the 19th century, followed by the British after World War I, and then with the establishment of the state of Israel in 1948 (Abu Saad 1991). After the establishment of Israel, the Bedouin movement with their herds was severely limited because the state nationalized the land and restricted areas of access. Under these conditions, Bedouins have become dependent upon wage labor in the majority Jewish sector in order to support themselves.

### 1.2. Ecological Variability in Residence Types

In the early 1970s, the Israeli government instituted a policy to move the Bedouin into urban settlements (Falah 1983), building eight Bedouin towns (one of which has grown large enough to now be considered a city). Half of the Negev Bedouins now live in these settlements, while the other half live in what are termed “unrecognized villages”, as only the settlements are recognized by the government as official municipal entities. This settlement policy, as well as the limits on travel in traditional nomadic areas because of federalization of lands within Israel and the establishment of national borders which are forbidden to cross, has meant that a good part of the Bedouin community is urbanized, no longer nomadic or engaged in animal husbandry, and lives in multi-clan towns or multi-ethnic cities. Exposure to diverse clans in the towns lessens kinship intensity. Exposure to multiple ethnicities in the city represents an even more diverse environment and further reduces kinship intensity. One outcome of lower kinship intensity is greater individualism (Schulz et al. 2019).

By 1990, 45% of the 90,000 Bedouins in southern Israel (Maddrell and Al-Grinawi 1990) had settled in seven officially legal towns. The remaining 55% resisted relocation, living in spontaneously created settlements (with 45% living in wood/tin shack settlements and 10% living in traditional tents). At the time of our data collection, about 67% of the Bedouins, out of a total of approximately 230,000, were living in government-built municipalities and towns (Israel Central Bureau of Statistics 2015; see Loeff 2023). The Bedouin villages and communities outside of these towns are not officially recognized by the state. In these unrecognized villages, there is no municipal entity to provide services such as health, education, and physical planning. Therefore, they lack basic infrastructure and public utilities such as water, electricity, and landline telephones (Negev Center for Regional Development 2010). The Israeli Ministry of Education did build elementary schools in some of the unrecognized villages, and recently, there have been some high schools built. In places where there are no schools, children attend schools in nearby recognized towns. With this, schooling has increasingly become available, and today, education is compulsory through high school (age 17) (OECD 2023).

In the early 2000s, 11 such unrecognized Bedouin villages were officially recognized and turned into two municipal regions. More recently, Bedouin people have begun living in the city of Be’er Sheva, which, before the establishment of the state, was a small market town for the nomads. With the establishment of the State of Israel, Be’er Sheva was settled by Jewish immigrants. When our data were collected, it had a population of over 200,000 (Israel Central Bureau of Statistics 2015); it is the government center of the Negev region of southern Israel. The Bedouin towns and villages, both recognized and unrecognized, are satellites of this urban center. Even the single-clan unrecognized villages are part of an increasingly urban area and have become part of an established official regional district. One of the eight towns established by the government now has a population large enough that it is officially classified as a city. Therefore, most Bedouins today are growing up in notably more urban environments than prior generations.

The three types of residences among the Bedouin—unrecognized villages, recognized towns, and mixed cities—represent different social ecologies. One distinguishing characteristic is exposure to diversity in terms of population, economic activity, values, and sources of knowledge. The unrecognized villages are homogenous, consisting of single clans with marriage permitted only within the clan and little division of labor. Hence, they have high kinship intensity. Recognized towns and the recognized Bedouin city have multiple clans. This situation would be expected to lower their kinship intensity. Mixed cities have multiple ethnic groups, in which Bedouins are minorities. Whole Bedouin clans or extended families do not reside in the mixed cities.

Whereas animal husbandry is still part of the economy of the villages, more people now work in the wage economy, although there are cultural restrictions on women leaving home without a man. Given the lack of infrastructure, access to media and communications technology has been limited. The recognized towns consist of multiple clans, but they remain basically segregated by clan, and marriage is allowed only within the clans. Thus, while kinship intensity is reduced relative to the unrecognized single-clan villages, it is still quite high.

The towns have commercial centers, schools, and municipal services, as well as easier transportation access to the urban center, so there is greater contact with the Jewish, non-Bedouin majority population. Although it is less common among the Bedouin to live in the mixed, predominantly Jewish city of Be’er Sheva or other mixed cities, those who do are in an urban, diverse environment, and children attend multi-cultural schools. These exposures should further reduce kinship intensity.

In sum, throughout the Anthropocene, the Bedouin of Israel have been forced to adapt to increasingly diverse and urbanized environments. However, the unrecognized villages, towns, and city represent a range of diversity from least to most. In our research design, we used historical variability to compare generations and current residential variability to compare groups living in different types of community.

#### Expected Epistemic and Identity Variability Across Residence Types

We thought that the greater diversity of towns compared with unrecognized villages and the still greater diversity of the city would lead to epistemic differences where residents of Be’er Sheva and other mixed cities would express the most reliance on self and experts as sources of knowledge and would have the weakest clan identity. Correspondingly, we thought that residents of the unrecognized villages would be the most reliant on family authority in their epistemic thinking and have the strongest clan identity. We thought that residents of the recognized towns and villages would fall in the middle in both of these psychological attributes.

### 1.3. Ecological Differences Among the Generations

The grandmothers in our sample grew up in the period before the transformation to more urban, multi-clan settlements. The mothers, if not actually living in the settlements, grew up in a period in which there was increasing regional urbanization and access to a growing urban center and with greater educational opportunity. The daughters, even if not living in one of the recognized towns or the mixed city, have grown up with greater participation in the commercial life of the urban center and improved transportation, including more widespread opportunity for women to have driver’s licenses.

One particularly important ecological shift across the generations is exposure to more diverse media (e.g., access to international television) and access to communications technology. Cell phones have become ubiquitous in all residence types. Based on our research among three generations of Northern Arabs in Israel and three cohorts of Romanians, increased exposure to diverse points of view through media leads to an epistemology in which the individual is seen as a valid source of knowledge and opinions (Ionescu et al. 2023; Weinstock 2015). International television also presents a diverse social world, thus presenting possible identities outside the family and clan.

Another important ecological shift is formal education. Only the daughters and mothers have grown up with official mandatory education through high school, although for the mothers, schools were much less accessible. In the daughters’ generation, there are more high schools closer to where they live, so in effect, more of the daughters are educated through high school. The mothers had variable educational opportunities, mainly depending on their place of residence, so some were educated through high school, and some were not. Because of the lack of educational facilities near their residences and the lack of accessibility, one choice was to send them to a neighboring village or the large city of Rahat, which had different clans. The parents often did not agree to this. There was no Bedouin school education when the grandmothers were young. That generation received informal education at home in life skills in the desert, and many at the time of our data collection were illiterate.

#### 1.3.1. Expected Epistemic Change Across the Generations

Among the psychological changes we expected to see between generations with such profound social change (Abu Aleon et al. 2019; Allassad Alhuzail 2018, 2022; Manago 2014) were shifts in epistemic perspective (Weinstock 2015) and identity (Abu-Rabia-Queder 2008; Allassad Alhuzail 2023a, 2023b). Given the demands of survival in an unforgiving environment with a subsistence economy and little contact with diversity, accepting the authority of clan leaders and the need to act as a unified collective would sculpt the epistemic perspective of the oldest generation.

In contrast, during the Anthropocene, handling encounters with diverse populations, developing skills related to wage labor, and formal education would lead to recognizing the individual in various contexts as a valid source of knowledge and thus a more individualistic conceptualization of knowledge (El-sana et al. 2024). Formal education would also lead to the recognition that specialized, impersonal, professional knowledge might be more appropriate in some instances than authoritative, community knowledge (Weinstock 2015).

#### 1.3.2. Expected Identity Change Across the Generations

Another psychological change we expected would be a shift in identity. We are using the term identity to mean a set of components through which one defines oneself for oneself and for the society in which one lives. The identity of the individual is personal because it expresses the point of view of the individual in relation to oneself and in relation to groups to which one identifies completely or partially identifies. The aspects of identity in relation to one’s society which are most relevant to the current study are the clan, extended family (sometimes termed a sub-family), and place of residence. We assumed that those growing up in a small-scale, single-clan environment, more characteristic of the grandmother’s generation, would express their identity in relation to the clan and extended family. Notably, a part of a Bedouin’s name includes the clan name, so a Bedouin’s clan membership is readily identifiable to others. In contrast, we expected that those growing up in more urban environments and with more contact with multi-clan or multi-cultural environments, which is more characteristic of younger generations, would express their identity in relation to the nuclear family or the place of residence.

Moreover, we expected that those with a more individualistic epistemic perspective would tend not to identify primarily as part of a clan. Epistemic shifts across the generations might loosen the power of social norms, thus allowing for shifts away from the traditional clan identity (Weinstock 2015).

### 1.4. Hypotheses

#### 1.4.1. Epistemic Shifts Across the Generations

**H1.**  *Younger generations will shift toward a more Gesellschaft-adapted epistemology in which the self and educated experts rather than family elders or religious authority are seen as valid sources of knowledge*.

#### 1.4.2. Identity Shifts Across the Generations

**H2a.**  *Younger generations will shift away from clan-based and extended-family-based identities*.

**H2b.**  *Younger generations will shift toward location-based identities.*

**H2c.**  *The adolescent generation will have the strongest local identity, and the grandmothers’ generation will have the strongest clan identity*.

#### 1.4.3. Modeling the Decline of Clan Identity Across the Generations

**H3a.**  *The role of epistemic perspective. We hypothesize that epistemic perspective will mediate the relation between generation and identity orientation such that the more Gesellschaft-adapted epistemology of the younger generation will make them more likely to adopt a local identity and less likely to adopt an extended-family or clan identity*.

**H3b.**  *The role of parents’ education. Education removes important expertise from the family and locates it in the broader society of teachers and books; hence, it reduces the authority of family elders. Hence, we predict that parents’ education is one mechanism by which younger generations develop weaker clan and extended family identities*.

**H3c.**  *The role of media use. We anticipated that media use would increase with every generation. Because the media use variable reflects exposure to authoritative ideas from outside the family, we thought that increased media use would weaken clan-based authority and would therefore serve as a mechanism leading to weaker clan and extended family identities, but stronger local identity*.

#### 1.4.4. Identity Variation Across Residence Types

**H4.**  *Because of variation in kinship intensity and ethnic diversity in the three residence types, we expected the greatest clan and extended family identity in the unrecognized villages; the greatest local identity in the mixed cities of Be’er Sheva, Jerusalem, Hebron, and a mostly Jewish town, Lehavim; and intermediate values in the recognized Bedouin towns and city*.

#### 1.4.5. Hypothesis 5: Epistemic Variability Across Residence Types

**H5.**  *Because of variation in kinship intensity and ethnic diversity in the three residence types, we expected more Gesellschaft-adapted epistemology (combining reliance on personal experience and educated experts as valued sources of knowledge) in the mixed cities, more Gemeinschaft-adapted epistemology (relying on the knowledge of family elders and devaluing school learning) in the unrecognized villages, and intermediate values in the recognized towns and city*.

## 2. Materials and Methods

### 2.1. Participants

The all-female sample consisted of 60 three-generation families of Israeli Bedouins living in the Negev Desert. That is, there were 60 adolescent girls (*M_age* = 16.18, *SD* = 1.10, range 13–19, all in high school), 60 mothers of these adolescent girls (*M_age* = 41.97, *SD* = 4.65, range 34–55), and 60 grandmothers of these adolescent girls (*M_age* = 67.43, *SD* = 6.08, range 56–83). In addition, the sample was drawn to be balanced across residence type: 20 families from unrecognized villages; 20 from a recognized Bedouin village, town, or city; and 20 from a city with a mixed population. Most of the participant families from mixed cities lived in Be’er Sheva, which has a majority Jewish population. No males were included in the sample because cultural norms required that interviewers and interviewees needed to be the same gender, and no male research assistants were available to do interviews in some of the locations. All the research assistants were Bedouin; they conducted interviews in the local Arabic dialect.

### 2.2. Materials and Variables

#### 2.2.1. Epistemic Perspective

Following other research on the effect of social change on values (Manago 2014) and epistemology (Weinstock 2015), epistemic perspective was assessed with the use of vignettes in which one character expresses the value of knowledge based on family authority or devalues academic learning to satisfy individual curiosity (Gemeinschaft perspective), and a second character expresses the value of impersonal professional knowledge or accepts individual interest in learning as a legitimate motivation for schooling (Gesellschaft perspective). The participants were asked which character they agreed with. Those endorsing the Gemeinschaft view were scored 1, while those endorsing the Gesellschaft view were scored 3. Those expressing their support for both views were scored 2. There were three vignettes, so the epistemic perspective score represented the average across the vignettes.

The topics of the vignettes concerned (1) the legitimacy of professional vs. traditional elder-based knowledge, (2) whether school is valued for an individual to gain knowledge or devalued because it does not contribute to performing an adult family role, and (3) the legitimacy of professional expertise vs. family as the source of knowledge. Appendix A contains the three vignettes in English and the original Arabic.

To verify that a single variable could be reliably created from the dilemmas, we checked if they were correlated with each other in the same direction using Spearman’s rank correlations. This procedure is the same as used in previous research (Abu Aleon et al. 2019; Weinstock et al. 2015). As shown in Table 1, all the dilemmas were correlated with each other. Because values can be situation-specific (Greenfield and Quiroz 2013), we did not expect a complete set of strong correlations.

#### 2.2.2. Identity

To assess the degree to which one expressed a local, clan, or family identity, the participants were asked to rate on a scale of 1 (disagree)–5 (agree) their level agreement with the following questions:

Local Identity: Does the term “[residence locale name]” fit your identity? (For example, “Does the term ‘Rahtawi’ fit your identity?” was asked of people living in the Bedouin city of Rahat.)

Clan Identity: Does the term “[clan name]” fit your identity? (For example, “Does the term ‘Abu-Quaider’ fit your identity?” was asked of people from the clan of that name.)

Family Identity: Does “[family name]” fit your identity? (For example, “Does the term ‘Hilali’ fit your identity?” was asked of people with that sub-family [extended family] name.)

#### 2.2.3. Sociodemographic Characteristics

##### Parents’ Education

The mother’s and father’s education were combined into a single 5-level variable. It consisted of (0) neither parent had formal education, (1) one parent had some formal education (primary or secondary), (2) both parents had formal education, (3) one parent had higher education, and (4) both parents had higher education.

##### Media Technology and Use

A media use variable was created which reflected exposure to content from diverse cultures or perspectives. The participants were asked what type of television they watched—Arab language, Hebrew language, or international programing—and to estimate how many hours or minutes of each they watched per day. They were also asked how much time each day they used social media sites on phones or computers. The media use variable consisted of the mean time they watched non-Arabic television and used social media in a day.

## 3. Results

### 3.1. Parent Education and Media Use: Descriptive Statistics

Table 2 shows that both parental education and media use increased with each generation. All generational differences were found to be statistically (parents’ education, *F*(1, 109.49) = 90.73, *p* < .001; media use, *F*(1, 104.68) = 16.91, *p* < .001). The Welch test was used because the generations had unequal variances. The Tamhane post hoc test, used when equal variances cannot be assumed, showed that parents of the adolescents had significantly more education than the parents of either the grandmothers (*p* < .001) or the mothers (*p* < .001) and that the mothers’ parents were more educated than the grandmothers’ parents (*p* < .05). The Tamhane post hoc test indicated that the adolescents’ media use was higher than that of both the grandmothers (*p* < .001) and mothers (*p* < .001), but there was no significant difference in media use between the grandmothers and mothers.

### 3.2. Correlations Among Generation, Epistemic Perspective, Parents’ Education, Media Use, Residence, and Identity Type

Before testing the hypotheses, correlations were assessed among the variables. As can be seen in Table 3, generation was correlated with each of the other variables such that the younger the generation, the more Gesellschaft-adapted the epistemology, the more reports of a local identity, and the fewer reports of a clan or extended family identity. In terms of epistemic perspective, Gesellschaft-adapted epistemology was negatively related to clan identity and positively related to parental education and media use. Residence in a more diverse location was positively correlated with parents’ education and media use and negatively correlated with extended-family identity. Parents’ education was positively correlated with media use and local identity, negatively correlated with clan identity and extended-family identity. Media use was positively correlated with local identity and negatively correlated with clan and extended family identity. All these correlations are supportive of the various hypotheses. We now turn to specific tests of the hypotheses.

### 3.3. Hypotheses Regarding Generational Differences in Epistemic Perspective and Identity Types

#### 3.3.1. Hypothesis 1: The Younger the Generation, the More Personal and Expert-Oriented the Epistemic Perspective (Adaptation to a Gesellschaft Ecology)

This hypothesis was confirmed. Our ANOVA showed significant differences in epistemic perspective by generation (*F*(2, 106.2) = 23.93, *p* < .001). Welch’s ANOVA was used because the generations had unequal variances. The younger the generation, the higher the mean vignette score (adolescents: *M* = 2.81, *SD* = 0.34; mothers: *M* = 2.52, *SD* = 0.58; grandmothers: *M* = 2.16, *SD* = 0.66), indicating that with each later generation, epistemic perspective became more adapted to a Gesellschaft ecology. Tamhane post hoc tests showed significant mean differences between grandmothers and mothers (*p* < .01), grandmothers and adolescents (*p* < .001), and mothers and adolescents (*p* < .01). Notably, the mean of the adolescent generation approached the ceiling of 3, and with a standard deviation of 0.34, the lower scores in the adolescent generation were around 2.5, indicating a decisive Gesellschaft orientation.

#### 3.3.2. Hypothesis 2a: The Younger the Generation, the Less Likely to Have a Clan Identity

This hypothesis was also confirmed. Our ANOVA showed significant differences in clan identity by generation (*F*(2, 115.3) = 4.19, *p* < .05). Welch’s ANOVA was used because the generations had unequal variances. As can be seen in Table 4, the grandmothers had the highest mean agreement with having a clan identity followed in turn by the mothers and the adolescent daughters. The Tamhane post hoc test found the only significant difference to be between the grandmothers and the adolescents (*p* < .05) such that the grandmothers had a significantly higher mean clan identity. The mothers’ mean clan identity was lower than that of the grandmothers and higher than that of the adolescents, but the means did not differ significantly.

#### 3.3.3. Hypothesis 2b: The Younger the Generation, the Less Likely to Have an Extended Family Identity

Although the order of means (Table 4) was in the predicted direction, our ANOVA showed no difference in extended-family identity by generation (*F*(2, 176) = 1.81, *ns*).

#### 3.3.4. Hypothesis 2c: The Younger the Generation, the More Likely to Have a Local Identity

Our ANOVA showed significant differences in local identity by generation (*F*(2, 114.5) = 8.07, *p* < .000). Welch’s ANOVA was used because the generations had unequal variances. As can be seen in Table 4, the adolescents had the highest mean agreement with having a local identity followed in turn by the mothers and the grandmothers. The Tamhane post hoc test found the only significant difference to be between the grandmothers and the adolescents (*p* < .001) such that the adolescents had a significantly higher mean local identity. The mothers’ mean local identity was higher than that of the grandmothers and lower than that of the adolescents, but the means did not differ significantly.

#### 3.3.5. Hypothesis 2d: For Adolescents, Local Identity Would Be Stronger than Extended-Family or Clan Identity; For Grandmothers, Clan Identity Would Be Strongest

This hypothesis was confirmed for both adolescents and grandmothers. Paired sample *t*-tests within each generation showed that for the adolescent generation, both clan identity and extended-family identity were significantly weaker than local identity (*t*(59) = −3.34, *p* < .001; *t*(59) = −3.30, *p* < .01, respectively). There was no difference between clan and extended-family identity (*t*(59) = 1.43, *ns*). As can be seen in Table 4, the mean score of local identity was higher than the mean of the other two identity types.

As for the grandmothers, Table 4 shows that, as predicted, clan identity was strongest. It was significantly stronger than extended-family identity (*t*(58) = 2.06, *p* < .05) and significantly stronger than local identity (*t*(57) = 5.21, *p* < .001).

#### 3.3.6. Identity Types by Mothers’ Generation

We had no hypotheses about differences in mothers’ identity types because we had no bases to expect differences. Indeed, we found no significant differences between any of the identity types in the mothers’ generation (clan/local: (*t*(59) = 0.09, *ns*); clan/family: (*t*(59) = 0.94, *ns*); local/family: (*t*(59) = 0.71, *ns*)).

### 3.4. Tests of Variables Mediating the Relationship Between Generation and Each Type of Identity

#### 3.4.1. Hypothesis 3a: Epistemic Perspective Mediates the Relationship Between Generation and Identity

**Figure 1:**
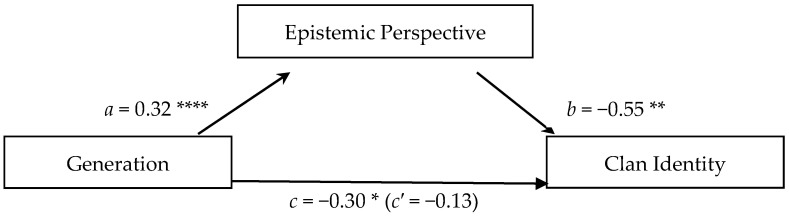
Test of the indirect effect of generation on clan identity mediated by epistemic perspective. *Note*: Significant negative coefficients in predictions of clan identity indicate that (*c*) the younger the generation, the less likely to have a clan identity, when including epistemic perspective as a separate variable in the model, and (*b*) the more personal and expert-oriented the epistemic perspective, the less likely to have a clan identity. The significant positive coefficients indicate that (*a*) the younger the generation, the more likely that one would have a personal and expert-oriented epistemic perspective. That the total effect (*c*) is significant, and the direct effect of generation (*c′*) is not significant when taking into account epistemic perspective as a mediator suggests that the correlation found between generation and clan identity could be explained in part by the mediation of epistemic perspective. This is confirmed by the significant indirect effect of generation through epistemic perspective (*CI* = [−0.289, −0.078]) on clan identity. * *p* < .05; ** *p* < .01; **** *p* < .001.

As epistemology was found to be significantly correlated with just clan identity (see Table 3); we tested just the hypothesis that epistemic perspective would mediate the relationship between generation and clan identity. The test for epistemology as a mediator of the relationship between generation and clan identity was performed using the bootstrapping method of PROCESS (v4.0, Hayes 2022). With this method, significance is determined if the confidence interval (*CI*) of the indirect effect of generation on clan identity through epistemic perspective does not include 0. Epistemic perspective did mediate this relation (*CI* = [−0.29, −0.08]). As shown in Figure 1, generation predicted epistemic perspective (*β* = 0.32, *p* < .001) such that the younger the generation, the more Gesellschaft-adapted the epistemology. Epistemic perspective then predicted clan identity such that those with a Gesellschaft perspective were less likely to have a clan identity (*β* = −0.55, *p* < .01). The total effect, that is, the relation between generation and clan identity without isolating epistemic perspective as a potential mediator (*β* = −0.30, *p* < .05), was significant so that the younger the generation, the less likely they were to have a clan identity. However, this relation became insignificant in the direct relation between generation and clan identity when eliminating the effect of epistemic perspective (*β* = −0.13, *ns*). This is a further indication that differences in epistemic perspective between the generations explain, at least in part, the generational differences in clan identity.

#### 3.4.2. Hypothesis 3b: Parents’ Education Mediates the Relationship Between Generation and Identity

**Figure 2:**
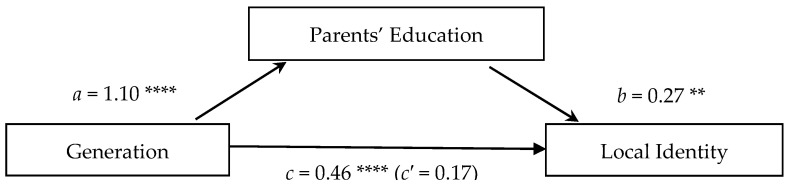
Test of the indirect effect of generation on the local identity mediated by parents’ education. *Note*: The significant positive coefficients in predictions of local identity indicate that (*a*) the younger the generation, the more likely that one would have parents with more education, (*b*) the more education the parents have, the more likely one is to have a local identity, and (*c*) the younger the generation, the more likely one is to have a local identity when not including parents’ education as a separate variable in the model. Whereas the total effect (c) is significant, the direct effect of generation eliminating any effect of parents’ education (*c′*) is not significant. This suggests that the correlation found between generation and local identity could be explained in part by the mediation of parents’ education. There are indirect effects of generation through parents’ education (*CI* = [0.126, 0.469]) on local identity. ** *p* < .01; **** *p* < .001.

In accord with our hypothesis, parents’ education did mediate the relation between generation and local identity (*CI* = [0.126, 0.469]). As shown in Figure 2, generation predicted parents’ education such that the younger the generation, the more formal education the parents had (*β* = 1.10, *p* < .001). Parents’ education then predicted local identity such that those with parents having more formal education were more likely to have a local identity (*β* = 0.27, *p* < .01). The relation between generation and local identity, without isolating parents’ education out as a potential mediator (*β* = 0.46, *p* < .001), was significant so that the younger the generation, the more likely they were to have a local identity. However, this relation became insignificant in the direct relation between generation and local identity when eliminating any effect of parents’ education (*β* = 0.17, *ns*). This is a further indication that differences in parents’ education between the generations explain, at least in part, the generational differences in local identity.

**Figure 3:**
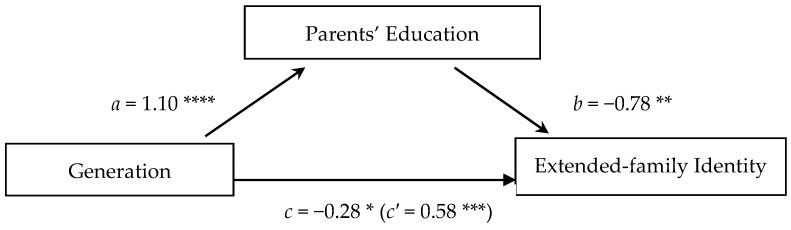
Test of the indirect effect of generation on the extended-family identity mediated by parents’ education. *Note*: The significant positive coefficients in predictions of extended-family identity indicate that (*a*) the younger the generation, the more likely that one would have parents with more education, and (*c′*) the younger the generation, the more likely one is to have an extended-family identity, eliminating any effect of parents’ education The significant negative coefficients indicate that, on their own (*b*), the more education the parents have, the less likely one is to have an extended-family identity, and (*c*) the younger the generation, the less likely one is to have an extended-family identity when not treating parents’ education as a separate variable. This means that the total effect is such that (*c*) the younger the generation, the less likely one would have an extended-family identity if the effect of parents’ education is not isolated, but the direct effect of generation when holding parents’ education constant (*c′*) has the opposite effect, so if parents’ education is removed from the equation, then the younger the generation, the more likely it is someone would express an extended-family identity. The indirect effects of generation through parents’ education on extended-family identity (*CI* = [−0.574, −0.354]) indicate that the negative correlation between generation and extended-family identity in the total effect is explained at least in part by the fact that those in the younger generation tend to have more educated parents. * *p* < .05; ** *p* < .01; *** *p* < .001; **** *p* < .001.

Parents’ education also mediated the relation between generation and extended-family identity (*CI* = [−0.574, −0.354]). As shown in Figure 3 and described in the models with the other two identity types, generation predicted parents’ education (*β* = 1.10, *p* < .001). Parents’ education then predicted extended-family identity such that those with parents having more formal education were less likely to have an extended-family identity (*β* = −0.78, *p* < .01). The relation between generation and extended-family identity, without isolating parents’ education out as a potential mediator (*β* = −0.28, *p* < .05), was significant such that the younger the generation, the less likely they were to have an extended-family identity. This relation was still significant, but in the opposite direction when eliminating any effect of parents’ education (*β* = 0.58, *p* < .001). This pattern indicates that negative correlation between generation and extended-family identity can be explained in large part by parents’ education.

Parents’ education did not predict clan identity (*β* = −0.16, *ns*), nor did it mediate the relation between generation and clan identity (*CI* = [−0.396, 0.048]).

#### 3.4.3. Hypothesis 3c: Media Use Mediates the Relationship Between Generation and Identity

**Figure 4:**
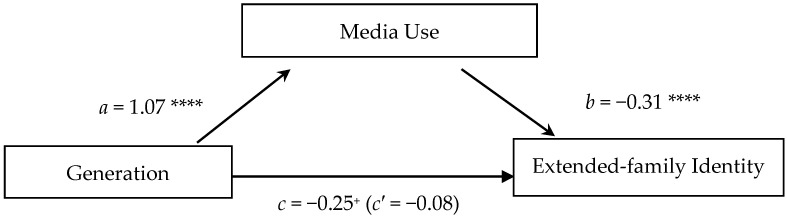
Test of the indirect effect of generation on the extended-family identity mediated by media use. *Note*: The significant positive coefficient in the prediction of extended-family identity indicates that (*a*) the younger the generation, the greater the media use. The negative coefficients indicate that (*b*) the greater the media use, the less likely one is to have an extended-family identity, and (*c*) the younger the generation, the less likely one is to have an extended-family identity when including media use as a separate variable in the model. When eliminating any effect of media use (*c′*), the relation between generation and extended-family identity disappears. This pattern suggests that media use accounts, at least in part, for the fact that younger generations identify less with their extended families. This apparent mediation is confirmed by the significant indirect effect of generation through media use (*CI* = [−0.480, −0.204]). ^+^ *p* = .077; **** *p* < .001.

Media use mediated the relation between generation and extended-family identity (*CI* = [−0.480, −0.204]). As shown in Figure 4, higher media use predicted weaker extended-family identity (*β* = −0.34, *p* < .001). Generation only marginally predicted extended-family identity (*β* = −0.25, *p* = .077), but the relation was notably weaker when eliminating any effect of media use (*β* = −0.08, *ns*). In the indirect effect, the fact that younger generations less frequently expressed an extended-family identity was explained mainly by their greater media use.

Contrary to the hypothesis, media use was not found to mediate the relation between generation and either clan identity (*CI* = [−0.263, 0.036]) or local identity (*CI* = [−0.093, 0.156]).

### 3.5. Hypothesis 4: Identity Variation Across Residence Types

Because of variation in kinship intensity and ethnic diversity in the three residence types, we expected the greatest clan and extended family identity in the unrecognized villages, the greatest local identity in the mixed cities (Be’er Sheva, Jerusalem, Hebron, and a mostly Jewish town, Lehavim), and intermediate values in the recognized towns. Table 5 shows the mean scores of each type of identity by place of residence.

A bootstrapped MANOVA showed that, overall, residence type significantly affected identity (Wilk’s lambda = 0.626, *F*(6, 346) = 15.23, *p* < .001, *η_p_*^2^= 0.21). The significant effects of residence type were concentrated in local identity (*F*(2, 175) = 4.13, *p* = .018, *η_p_*^2^ = 0.05) and extended-family identity (*F*(2, 175) = 47.541, *p* < .001, *η_p_*^2^ = 0.35). Not quite as predicted, the greatest extended-family identity was found in both the recognized and unrecognized villages, and these results were essentially identical (*M* = 4.37 and *M* = 4.38 respectively), but, as predicted, extended-family identity was significantly weaker in the cities (*M* = 2.44) than in either the recognized towns (Tamhane test, *p* < .001) or the unrecognized villages (Tamhane test, *p* < .001).

As predicted, the greatest local identity was found in the mixed cities (*M* = 4.19). Local identity was significantly stronger in the mixed cities than in the recognized towns (*M* = 3.54) (Tamhane test, *p* = .016). Although the difference between local identity in the cities and the unrecognized villages (*M* = 4.02) was in the predicted direction, it was not statistically significant.

Clan identity, as predicted, was greatest in the unrecognized villages and weakest in the cities. However, these differences were not large enough to attain statistical significance.

The ordering of mean identity within each residence type can be seen in Table 5 by comparing the columns in each row. Paired-sample *t*-tests comparing the columns found no significant differences between the means of the identity types in the unrecognized villages (clan v. local identity, *t*(59) = 1.12, *ns*; clan v. family identity, *t*(59) = 1.04, *ns*; local v. extended family identity, *t*(59) = 1.68, *ns*). In the recognized towns, local identity was the weakest, with significant differences between local identity and both clan identity (*t*(58) = 3.40, *p* = .001) and extended-family identity (*t*(57) = 4.47, *p* < .001). Clan and extended-family identity did not differ (*t*(59) = 1.51, *ns*). In the mixed cities, local identity had the highest mean, which differed significantly from the mean of extended-family identity (*t*(58) = 5.32, *p* < .001) but not from the mean of clan identity (*t*(58) = 1.14, *ns*). Clan identity was stronger than extended-family identity (*t*(58) = 6.03, *p* < .001).

### 3.6. Hypothesis 5: Epistemic Variability Across Residence Types

Because of variation in kinship intensity and ethnic diversity in the three residence types, we expected greatest Gesellschaft orientation in the mixed cities, greatest Gemeinschaft orientation in the unrecognized villages, and intermediate values in the recognized towns and villages. However, the one-way ANOVA using the Welch test, because there were unequal variances between the residence types, showed no difference in epistemic thinking across residence types (*F*(2, 115.45) = 0.087, *ns*).

## 4. Discussion

As predicted, the infusion of education and media across the generations weakened traditional authorities as sources of knowledge leading to a more personal and expert-oriented epistemology. This epistemic perspective, in turn, weakened clan identity. Formal education also weakened extended-family identity. Both formal education and mixed-city residence promoted local identity. In contrast, residence in a recognized town reduced local identity.

This last result is in line with Allassad Alhuzail’s (2023a) finding with young professional men living in recognized towns. She summarizes her findings by the title of her article, “I just live in the village, but I don’t belong to it”. Her interviews indicated that a major reason for the lack of identity with recognized towns was the lack of services, facilities, shops, and entertainment compared to the urban environments of Israel’s mixed cities, including men’s professional environments. Although the young professional men in that study constitute a different population in terms of gender, age, and educational achievement from the one reported here, their perspective could shed light on why local identity was significantly greater for families residing in mixed cities in our study compared to recognized towns. Also in line with Allassad Alhuzail’s finding that these men did identify with their clans, in the current study, the women in the recognized towns identified significantly more with their clans than their localities. It is also of note that we did not find a significant difference in local identity between mixed-city residents and residents of unrecognized villages, and there was not a significant difference between clan and local identity among the women from the unrecognized villages. The reason for this is undoubtedly that the unrecognized villages are within the ancestral lands of their residents, and so residents are attached to the land, despite the lack of facilities or services. This stands in contrast with the Bedouins who have been forced into the recognized towns, with little regard for their traditional locations or respect for the traditional geographical separation of each clan.

The finding that epistemic perspective mediates the generational shifts away from clan identity suggests that holding a view that knowledge is not held exclusively by social authorities in the community loosens the traditional ties to the clan. In the larger picture, the implication that the recognition and acceptance of individual and expert perspectives is adaptive to the shift to Gesellschaft social ecologies would seem to fly in the face of recent history, which has seen a move toward authoritarian perspectives and against professional expertise. There are several possible explanations for this trend which are not inconsistent with sociodemographic shifts toward Gesellschaft ecologies. One explanation is that the loosening of traditional external authority and certainty can represent a threat to well-being. Perry (1970), in his seminal research on epistemic development, said that students spoke of their creeping realization that knowledge was uncertain and open to multiple perspectives as a crisis which shook their faith not just in deciding how they could know but how they should live. Thus, we see in the very shift which loosens the hold of an absolutist, authoritative world the possible seeds of embracing authoritarianism and rejection of expert, but impersonal science, such as we have increasingly seen in much of the world today—for example, in the rise of misinformation and populist movements. Perhaps this will happen to the Bedouins at a later point in their history.

Another related possibility is that the political variable of democracy also needs to be considered as part of a Gesellschaft environment. Note that “democratic” is part of the WEIRD (Western, Educated, Industrial, Rich, and Democratic) formulation (Henrich et al. 2010), factors that are very related to Gesellschaft features. If we consider democracy to be, on the political level, a component of a Gesellschaft ecology, then we could also conclude that the movement away from democracy and toward dictatorship represents a Gemeinschaft political force. That this factor would lead to a cognitive shift toward respect for authority and distrust of scientific expertise would be very much in line with the theoretical analysis presented here. Unfortunately, we cannot evaluate this possibility, because our data were collected before the right-wing political movement toward dictatorship had become evident. Perhaps the Bedouins of today, who have grown up after our data collection and are living with a turn toward authoritarian government in Israel, may have reversed their epistemic direction and moved toward greater respect for traditional, elder-based knowledge, as well as knowledge that benefits family and community.

In conclusion, as of 2014–2015, when the data were collected, the diversity of opinions and perspectives characteristic of the Anthropocene through the explosion of media technologies, the expansion of formal education, and the growth of city living had made Bedouin thinking more individualistic and respectful of professional expertise while undercutting identification with the hierarchical kin-based organizations that constitute clans and extended families. At the same time, residence in Israel’s facility-filled mixed cities has augmented identification with these locations for their Bedouin residents.

## Figures and Tables

**Table 1:** Spearman’s rank correlations between epistemic perspective dilemmas.

	Professional vs. Elder-Based Knowledge	Value of Knowledge Contributing to the Individual vs. Family
Value of knowledge contributing to the individual vs. the family	0.35 ***	
Legitimacy of professional vs. family-based knowledge	0.30 ***	0.27 ***

*** *p* < .001, one-tailed.

**Table 2:** Means and standard deviations of levels of parents’ education and media use by generation.

Generations	Parents’ Education		Media Use	
	*M*	*SD*	*M*	*SD*
Grandmothers	0.36	0.74	0.47	1.48
Mothers	0.86	1.25	0.93	1.33
Adolescent girls	2.52	1.00	2.62	2.31

Note. Parents’ Education is the mean of the following scale: 0 = neither parent with formal education; 1 = one parent with some formal education; 2 = both parents with some formal education; 3 = one parent with higher education; 4 = both parents with higher education. Media Use figures are the number of hours per week on social media and watching non-Arabic television programs.

**Table 3:** Correlations among generation, epistemic perspective, parents’ education, media use, residence, and identity types.

	Generation ^a^	Residence ^b^	Epistemic Perspective	Parents’ Education	Media Use
Epistemic perspective	0.47 ***	0.04	-		
Parents’ education	0.67 ***	0.33 ***	0.31 ***	-	
Media use	0.54 ***	0.38 ***	0.17 *	0.59 ***	-
Local identity	0.31 ***	0.08	−0.04	0.36 ***	0.18 *
Clan identity	−0.19 *	−0.06	−0.29 ***	−0.24 **	−0.24 **
Extended-family identity	−0.14 ^+^	−0.49 ***	−0.01	−0.49 ***	−0.39 ***

^a^ Spearman’s rank correlations were tested between generation and the other variables. ^b^ Spearman’s rank correlations were tested between residence and the other variables. Residence was ordered by degree of diversity (unrecognized village, recognized town, and mixed city). ^+^ *p* = 0.64; * *p* < .05; ** *p* < .01; *** *p* < .001.

**Table 4:** Means and standard deviations of identity types by generation.

	Identity Type					
Generation	Clan Identity		Local Identity		Extended-Family Identity	
	*M*	*SD*	*M*	*SD*	*M*	*SD*
Grandmothers	4.44	1.06	3.43	1.40	4.00	1.49
Mothers	3.97	1.33	3.95	1.23	3.77	1.53
Adolescents	3.83	1.46	4.35	1.06	3.47	1.58

**Table 5:** Means and standard deviations of identity types by place of residence.

	Identity Type					
Generation	Clan Identity		Local Identity		Extended-Family Identity	
	*M*	*SD*	*M*	*SD*	*M*	*SD*
Unrecognized villages	4.18	1.36	4.02	1.32	4.38	1.17
Recognized towns	4.12	1.04	3.54	1.15	4.37	0.91
Mixed cities	3.92	1.51	4.19	1.31	2.44	1.58

## Data Availability

The raw data supporting the conclusions of this article will be made available by the authors on request.

## Appendix A

*The epistemic perspective scenarios (in English and the original Arabic)*

The legitimacy of professional vs. traditional, elder-based knowledge

Mahmoud and Fatima are a young couple. Fatima suffers from medical risks. The doctor told them that they must undergo treatment and take a medicine for a year before the birth of the child. But Mahmoud’s mother does not believe in modern medicine and only believes in the medicine prepared by the grandmother, and she pressures Fatima to give birth to the child and urges her to give birth as soon as possible.

Whom do you agree with or disagree with? Why?

Whether school is valued for gaining knowledge for individual enjoyment or devalued because it does not contribute to an adult family role.

Wafa goes to high school. She wants to continue to post-secondary education, but her parents believe that she should not continue and study just until the twelfth grade. They believe it is enough to be a good wife in the village. But Wafa likes to learn new things.

Whom do you agree with or disagree with? Why?

The legitimacy of professional vs. family-based knowledge.

Abdullah noticed that his son was having difficulties at school. He met with the school psychologist. After a number of meetings, they decided that his son, who is studying in the second grade, should study in a special education school. Abdullah agreed with the psychologist and decided to send his son to a special education school after a week. The grandfather objected to that, because the family knows more about what is suitable for their son than anyone else. The boy’s situation is good, and he should stay in the regular school. Abdallah claims that the psychologist understands better than they do in such a situation.

Whom do you agree with or disagree with? Why?

شرعية المعرفة المهنية أو التقليدية المستندة إلى كبار السن
محمود وفاطمة هما زوجان شابان. تعاني فاطمة من مخاطر طبيّة. قال لهما الطبيب بأنه يجب عليهما أن يخضعا للعلاج وتناول الدواء لمدة عام قبل ولادة الطفل. ولكن والدة محمود لا تؤمن بالطب الحديث وتؤمن فقط بالدواء التي تعده الجدة، وهي تضغط على فاطمة كي تنجب الطفل وتحثها على الولادة في أقرب فرصة ممكنة.
لمَن يجب الاصغاء؟ لماذا؟

هل يتم تقدير المدرسة لاكتساب الفرد للمعرفة من أجل المعرفة ذاتها؟
قيمة التعليم والمعرفة: البنت تذهب للمدرسة.
تريد وفاء الذهاب للمدرسة الثانويَّة، إلاّ أنَّ والديْها يعتقدانِ بأنّه يجبُ عليها أن لا تواصل تعليمها الثانويّ، وأن تتعلمّ حتى الصف الثاني عشر. ويعتقدون أن يكفي أن تكون زوجة صالحة في القرية. ولكنَّ وفاء تحبُّ أن تتعلّمَ أشياء جديدة.
ماذا تعتقد؟ مع من توافقون ولا توافقون؟ لماذا؟

الفرد أو الجماعة كمصدر للمعرفة.
لاحظ عبد الله أن ابنه يواجه صعوباتٍ في المدرسة. التقى مع الأخصائيّ النّفسي في المدرسة. بعد عددٍ من اللقاءات قرّرا أنَّ ابنَه الذي يتعلّم في الصف الثّاني يجبُ أن يتعلَّمَ في مدرسة للتربية الخاصّة. وافق عبد الله مع الأخصائي النّفسي وقرّرا إرسال ابنه بعد أسبوع إلى مدرسة للتربية الخاصّة. اعترض الجدُّ على ذلك، بالنسبة لهم فهم أدرى بما هو ملائم لابنهم أكثر من أيّ شخصٍ آخرَ. وضعُ الولد جيّد ويجب أن يبقى في المدرسة العادية. يدعي عبد الله أن الأخصائي النفسي يفهم أفضل منا في مثل هذه الحالة.
أي منهم صاحب الموقف الصحيح؟ مع من توافقون ولا توافقون؟ لماذا؟
